# Mendelian Randomization and Infection: Pitfalls and Promises

**DOI:** 10.1093/infdis/jiaf251

**Published:** 2025-05-28

**Authors:** Fergus Hamilton, Guillaume Butler-Laporte, George Davey Smith

**Affiliations:** Medical Research Council Integrative Epidemiology Unit, University of Bristol, Bristol, United Kingdom; Infection Science, North Bristol NHS Trust, Bristol, United Kingdom; Division of Infectious Diseases, McGill University Health Centre, Montreal, Quebec, Canada; Lady Davis Institute for Medical Research, Jewish General Hospital, Montréal, Québec, Canada; Medical Research Council Integrative Epidemiology Unit, University of Bristol, Bristol, United Kingdom

**Keywords:** Mendelian randomization, genetics, malaria, epidemiology, causal inference

## Abstract

Mendelian randomization (MR) is an increasingly common study design in infectious diseases (ID). It holds promise for identifying causes and consequences of infections where conventional epidemiology has struggled, and can highlight plausible drug targets, as shown in successful coronavirus disease 2019 (COVID-19) trials (baricitinib, tocilizumab). However, many current applications provide limited insight due to violations of core assumptions, yielding uninterpretable results. This article reviews MR principles, assumptions, and specific challenges in ID. We highlight examples violating key assumptions, noting that MR studies using infection as an exposure are particularly prone to bias compared to using infection as an outcome. We discuss the future of MR in ID, emphasizing appropriate application to address causal questions unanswerable by other methods and capitalize on emerging opportunities where MR can provide unique insights.

## Teaser

Mendelian randomization (MR) offers promise for infectious disease research but faces challenges. This update reviews MR principles, pitfalls specific to infection studies, and future opportunities, emphasizing correct application to yield reliable causal insights and identify drug targets.

Mendelian randomization (MR) is now an established technique in etiological epidemiology. There has been undoubted success of this technique, with establishment of key risk factors for disease [1], identification of drug targets [2, 3], and—as importantly—clear evidence that certain risk factors are not causal for diseases [4–6].

**Figure 1.**
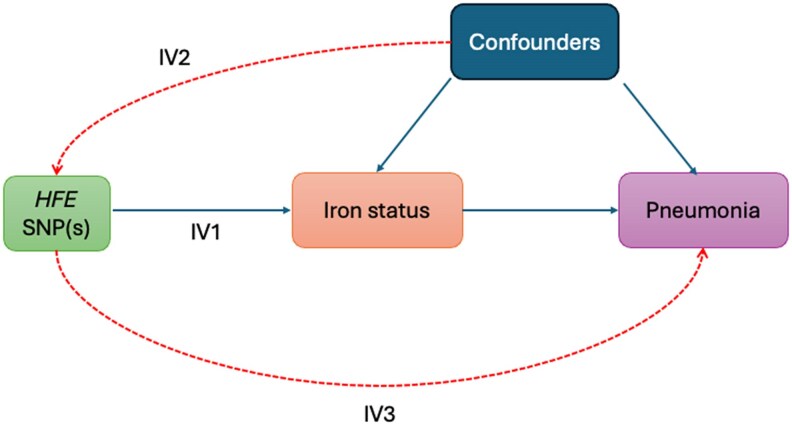
A directed acyclic graph describing Mendelian randomization. An illustrative example might be the use of genetic variants associated with iron levels as instruments to explore the causal effect of iron status on pneumonia risk. The 3 IV assumptions are also visualized: it is possible to test whether the instruments influence the exposure, but it is not possible to falsify whether the dashed arrows (IV2 and IV3) exist, although sensitivity analyses do exist. Abbreviations: IV, instrumental variable; SNP, single nucleotide polymorphism.

The essence of MR (described formally elsewhere [7–9]), is that genetic variation has certain unique properties that allow it, under certain assumptions (see Box 1), to be used to estimate causal effects of an exposure on an outcome (Figure 1), which are in principle unbiased by the confounding of exposure and outcomes, reverse causation, and related issues that plague observational studies [10, 11].

**Box 1. Key Assumptions and Study Design in Mendelian Randomization**

Mendelian randomization (MR) relies on a set of core assumptions that ensure genetic variants can be used as valid instruments for causal inference (Figure 1). These assumptions are critical in preventing bias from confounding, reverse causation, or other limitations that affect observational studies.

More formally, a genetic instrument must satisfy 3 key criteria:

1. Instrumental relevance: The genetic variant must be robustly associated with the exposure (eg, the genetic variants associated with iron status).
2. Exchangeability: The instrument should not be confounded with the outcome (ie, there are no factors influencing both the instrument and the outcome). This might be violated if, for example, lower rates of pneumonia occur in specific ancestries as well as a lower frequency of the *HFE* single-nucleotide polymorphism such as those with African ancestry
3. Exclusion restriction: The instrument affects the outcome only through the exposure and not via alternative pathways. This might be violated if the genetic variant has some other consequences unrelated to iron. For example, genetic variants in the interleukin 6 (IL-6) pathway alter infection risk and also alter iron levels, but the effect on infection risk is likely related to the IL-6 activity not iron levels.

Assumption 1 can be empirically tested, but assumptions 2 and 3 are unfalsifiable, making careful study design essential. The most critical assumption in MR studies, often referred to as the third instrumental variable (IV) assumption (exclusion restriction), requires that the association between a genetic variant and the outcome occurs exclusively through the hypothesized exposure. Violations of this assumption, such as pleiotropy (when a variant influences multiple traits), are a major concern in MR research and require sensitivity analyses to detect potential bias.

For infection-related MR studies, dynastic effects may also be relevant—genetic variants influencing parental traits (eg, maternal iron levels) could indirectly affect infection risk in offspring, leading to potential confounding. Additionally, pathogen exposure itself is not typically under strong genetic control, complicating MR studies that use infection as an exposure rather than an outcome. It is unlikely that there are many genetic variants that solely alter the risk of a single infection and so there are likely few MR studies that use infection as an exposure that do not violate the third IV assumption.

These factors highlight the importance of selecting instruments carefully and considering the unique challenges of applying MR in infectious disease research.

Perhaps surprisingly given the key role of infection in epidemiology, MR studies in infection were rare until around 5 years ago, with only 48 PubMed results for the search “Mendelian randomi[z/s]ation AND infection” up to 2018. However, since then, there has been an explosion of studies, with 747 papers. Some of these papers provide valuable causal insight—the MR analyses identifying expression of *TYK2* as causal for severe disease in the COVID-19 pandemic [12] directly led to identification of baricitinib as a potential and ultimately successful [13] drug—but many simply use publicly available data to address incoherent questions of little practical relevance [14]. This explosion of papers largely stems from the widespread public availability of summary genetic data.

**Box 2. How to perform a Mendelian Randomization study**

**Figure 2.**
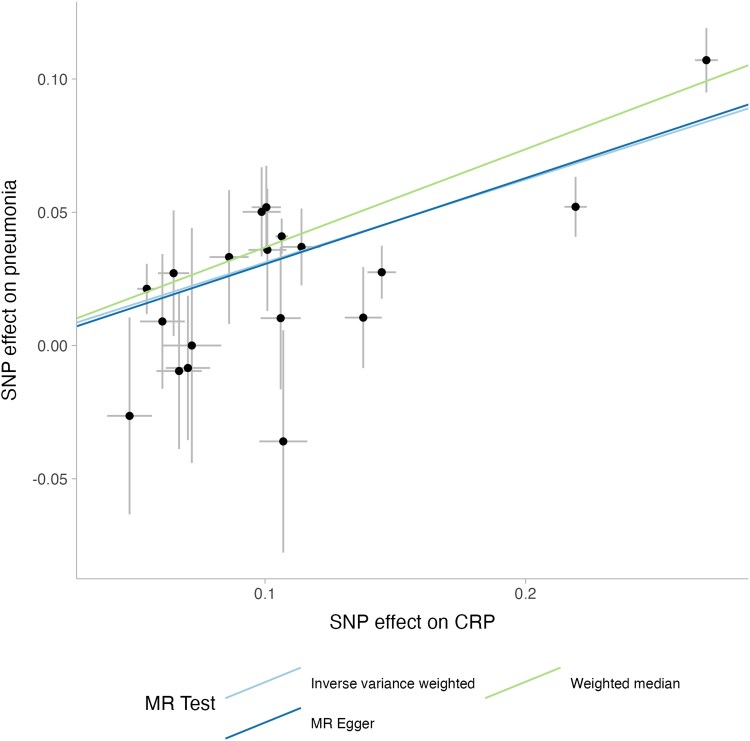
An example of a Mendelian randomization scatter plot. On the x-axis we plot the effect of the instrument on the exposure (in this case CRP), and on the y-axis we plot the effect on the outcome (pneumonia). The plotted lines represent meta-analyses of the individual SNPs, and the slope of each line represents the causal effect estimate. In the below figure, SNPs that increase CRP tend to also increase the odds of pneumonia, and there is therefore evidence of an effect of increasing CRP on the odds of pneumonia. Abbreviations: CRP, C-reactive protein; SNP, single nucleotide polymorphism. Error bars represent standard errors.

First, researchers should clarify the causal question in terms of exposure and outcome. Once that has been done, they should then identify genetic variants that reliably associate with the exposure (instruments). This can be done using individual-level data (eg, by performing a genome-wide association study [GWAS] of the exposure), or by accessing publicly available summary statistics (eg, at the Medical Research Council Integrative Epidemiology Unit OpenGWAS catalogue, or the European Bioinformatics Institute GWAS catalogue). Subsequently, researchers then try to identify the association between the instrument(s) and the outcome. Again, this can be done in individual-level data or using summary statistics. Finally, simple mathematical equations can be used to derive the estimated causal effect of the exposure on the outcome (Figure 2). For example, the commonest estimate is the Wald ratio, which is calculated by dividing the instrument-outcome association by the instrument-exposure association.

This procedure is often performed for multiple instruments (eg, multiple single-nucleotide polymorphism [SNPs]), and the results meta-analyzed to generate a summary estimate. Various approaches can be used to meta-analyze and try and account for outlier SNPs, for example. It is also critically important to ensure that the direction of the effects is the same for both the exposure and the outcome—numerous Mendelian randomization studies have accidentally got the direction of the effect wrong for 1 dataset, leading to results in the opposite causal direction.

More importantly, many of these analyses violate key MR assumptions [7, 15], making their conclusions invalid, or do not highlight enough the limitations of such analyses. Here, we highlight issues to consider in MR studies in infection, noting the recent retraction [16] of an MR article in infection due to a violation of the instrumental variable (IV) assumptions.

## TERMINOLOGY

There are 3 core components in an MR analysis: the exposure, the outcome, and the instrument(s). For the question: “does iron status alter the risk of pneumonia?” the exposure is the variable the researcher is interested in estimating the causal effect of (eg, iron status) and pneumonia would be the outcome. The instruments are genetic variants (usually single nucleotide polymorphisms, SNPs) that have an effect on the exposure. For example, variants in *HFE* that increase iron and lead to hereditary hemochromatosis could be used as instruments for the exposure of “iron status.” The key assumptions and an explanation of how an analysis is performed are available in Box 2 and Figure 2.

In the rest of the article, we now describe both general and specific issues pertaining to MR in infectious disease. First, we review the challenges of using infection itself as an exposure, then focus on the challenges of infection as an outcome and other general issues in MR.

## INFECTIONS AS AN EXPOSURE

Many studies attempt to use infection as an exposure in MR studies, which is often very challenging due to violations of key MR assumptions. In particular, we are aware of few genetic variants that strongly alter the incidence of any infectious disease, and for which we are confident of the biological process in which the genetic variant reduces incidence (variants in blood cell traits altering the risk of malaria being a notable exception [17–22]), and are also confident that this effect is specific to this one infection. As such, MR studies that aim to ask the causal question “does this infection alter the risk of this outcome” often use genetic variants that associate with some other feature of infection, such as antibody response to a pathogen [23, 24]. This has major issues. Firstly, as acquisition and susceptibility to an infection is a function of both immunological susceptibility (either innate or acquired) and pathogen encounter (often defined as pathogen exposure, we choose the term encounter here to differentiate from exposure as used in MR), you cannot easily use genetic variants that alter host response to an pathogen to answer the questions around whether being exposed to that pathogen causes a certain trait. Because antibodies can derive from infection or vaccination, caution is needed when inferring causality. Genetic variants associated solely with *vaccine*-induced antibody levels do not provide causal evidence linking the *infectious disease itself* to an outcome [25]. Secondly, nearly all of the genetic associations for antibody response are, unsurprisingly, in the HLA region of the human genome, which encodes the antibody response [23]. This is an extraordinarily complex region of the genome and has a number of challenges for genetic epidemiology, including high levels of linkage disequilibrium (nonzero correlation of multiple genetic variants) but also a very high risk of pleiotropy (when a genetic variant affects many different traits). It is much more challenging to fulfil the third IV assumption, although a method has recently been developed to test causality at this locus [26].

**Figure 3.**
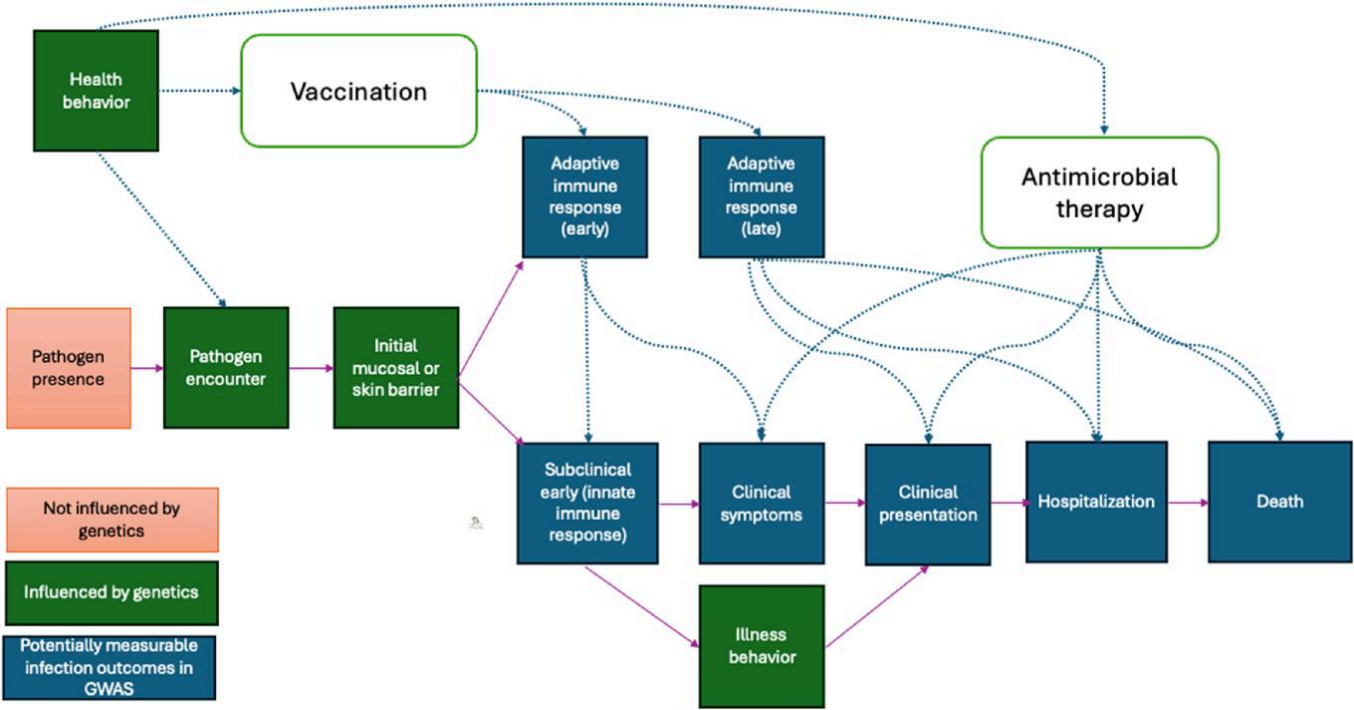
A noncomprehensive schematic of infection progression and potentially measurable outcomes in GWAS. The blue boxes represent outcomes of infection, all of which are potentially measurable in GWAS and themselves influenced by genetics. The orange box (pathogen presence) is independent of genotype, the green boxes represent plausible genetic influences that would not be measured as outcomes in infection GWAS but could clearly influence progression through the stages. This is clearly a simplification, and there is likely circularity in the diagram. However, the key feature is that when measuring, for example, hospitalization with infection, the person must have progressed through the stages before, and the genetic influences on each stage are highly likely to be different. Abbreviation: GWAS, genome-wide association study.

This underlines a major difference between infection traits and other traits—that the acquisition and progression of infection result from a more nuanced relationship between environment and genetics (see Figure 3 for schematic). Firstly, and obviously, in some settings pathogens are not present (eg, COVID-19 did not exist before 2019). Therefore, an MR study that uses outcome data from before 2019 clearly cannot measure the effect of “COVID-19 infection” on the outcome—COVID-19 did not exist, and reported effects *cannot* be due to COVID-19! However, such studies exist in the published literature [27, 28].

Pathogen encounters are generally not under strong genetic control. It appears for most infectious diseases transmitted from human to human, the major determinant of risk is driven by the environment and the giver, not the receiver (see, eg, “super-spreaders” in many infections [29]). This is an oversimplification: clearly the risk of acquiring, for example, sexually transmitted infections relates strongly to both giver, receiver, and environmental factors.

Although features like attitude to risk might influence pathogen encounters, this effect is likely small at the population level. Supporting this, the population-level prevalence of common infections differs widely (eg, seroprevalence of cytomegalovirus >90% aged 3 years in South Africa [30], approximately 60% aged 60 years in the UK [31]). This does not mean these effects are unimportant, but there are likely not strong individual variants that could be used as instruments in MR. Of course, vaccination (and by extension, genetics that alter the response to and/or uptake of vaccination) can influence the course of this infection (often dramatically), but vaccination does not alter whether or not one gets exposed to a pathogen, except by the second-order public health effects of vaccination, which are not driven by germline genetics.

As such, genetic susceptibility to pathogen acquisition is likely generally low, and reduced compared to genetic susceptibility to some disease progression traits (eg, symptomatology, requirement for treatment). Where we do have good data for this (malaria [32, 33] and COVID-19 [34]), heritability—broadly, the proportion of variance that can be explained by genotype—for incidence of disease appears to be lower than for severity (accepting that estimates of heritability are challenging [35]). As a final problem, pathogens evolve: either via random drift or more commonly in response to the numerous external stimuli (eg, immune response, ecological niches, antibiotics). And, in return, humans evolve over time: there is evidence supporting rapid change in both individual loci (eg, *LP*, the gene that encodes the lactase persistence phenotype [36, 37]), and polygenic signals (relating to adiposity) that appear to have been related to infection [36].

Therefore, a genome-wide association study (GWAS) of severe COVID-19 today would look very different from a GWAS performed in the prevaccine era (because we would essentially identify variants conditioning on vaccine failure). On a much longer timescale, we have strong evidence of coevolution between *Plasmodium falciparum* and humans over many thousands of years—the effect of any variant has to be understood in the context of that evolutionary history [38].

All of these issues suggest that MR studies that aim to ask the question whether acquisition of infection leads to an outcome are very challenging. We are aware of some well performed studies in this area—largely using malaria exploiting the sickle cell variant to uncover the effects of severe malaria on iron deficiency [39] and other outcomes [40–42]. It is important to note that the sickle variant does not influence malaria encounter, it simply makes survival of *Plasmodium* in humans more challenging, essentially reducing the severity of disease. One study exploited the lack of malaria in US populations (where there is no malaria) as a negative control [43] to confirm the effect of the variant was acting through reduction in malaria. This may be a promising approach where multiple populations with extreme differences in pathogen encounter are available.

## OTHER EXPOSURES

Before going on to describe the issues of infection as an outcome, it is worth highlighting other potential exposures and their foibles. We were invited to write this article due to our identification of the incorrect use of “drug receipt” as an exposure in a recently retracted paper [16]. These flawed study designs generally use genetic data associated with drug usage (or in the recent example the pharmacokinetics of the drug in question) as an instrument for drug usage (see, eg, [44, 45] for other examples). This approach does not make sense. In the case of the retracted paper, an analysis was performed purporting to use MR to assess whether ticagrelor alters the risk of infection. In this analysis, the authors made the claim that their genetic variants are instruments for “ticagrelor exposure” [43]. That is, that carrying 1 allele of the genetic variant increases the “exposure” relative to the other allele. The authors identify this variant from a GWAS that calculated the association between genetic variants and drug levels of ticagrelor in patients treated with ticagrelor in a randomized trial (PLATO [46]) [47]. This study had identified 2 “independent” genetic variations in the *CYP3A4* region that altered levels of ticagrelor, although they did not identify that carriers of these variants had any detectable effect on safety and efficacy of the drug.

Importantly, these are *not* valid instruments for “the effect of ticagrelor” in the general population. These genetic variants—as expected from their location in the cytochrome genes—alter the exposure of ticagrelor (and likely, other drugs) in those who are taking ticagrelor. They cannot increase the exposure of ticagrelor in those not taking the drug. Therefore, in the vast majority of people, these variants—if they have any effect—are certainly not doing it through altering the pharmacokinetics of ticagrelor, as participants are not taking ticagrelor. In essence, this study violates the first IV assumption: the variant does not associate with the actual exposure (“taking ticagrelor”) [16]. Given this implausibility and other issues, the paper was retracted. This is analogous to our discussions above: genetic variants that alter the probability of receiving ticagrelor are not the same as those that alter the effect of ticagrelor, as genetic variants that alter the probability of getting exposed to a pathogen are different to those that alter disease progression.

Other papers that violate this or the other IV assumptions remain unretracted; a study used genome-wide association studies of predicted PM2.5 (a marker of air pollution) based on home address as instruments to explore whether air pollution might be associated with longevity or cancer [48, 49]. This is clearly impossible: genetics cannot alter air pollution, and associations are likely driven by strong confounding between genetics and socioeconomic status, which itself correlates with air pollution.

## INFECTION AS AN OUTCOME

More commonly, infection traits are used as outcomes in MR studies. The question is asked whether some exposure is causally related to the infection outcome. Again, the definitions here of infection outcomes are critical (see Figure 3), and researchers should be careful to differentiate between a host-immune response, a clinical diagnosis of infection, or some other phenotype. Self-reported infection is commonly used, but it should be clear that many infections are ubiquitous, and therefore self-reported “upper-respiratory tract infection” may well represent those who recall such infections, rather than their presence or absence, as all humans suffer with upper respiratory tract infection. It is less clear what the role of antibody (or other) response as an outcome means, or when these MR studies add value—except clearly in the context of vaccination response where there is a known relationship between antibody status and clinical infection, and where genetic variants can be used to potentially help understand vaccine biology and/or improve vaccines.

However, this approach is a potentially fruitful area for MR, with numerous studies identifying causative factors, some unknown, some well known for either development of infection or for progression of that infection in some fashion [12, 50–57]. However, the complexity of interpretation of MR estimates can be highlighted by MR studies that use variation in the interleukin-6 receptor (IL6R) to proxy the effect of IL-6 inhibition [54–57]. This MR approach has been widely used and is notable for identifying a potential protective effect of IL-6 inhibition on cardiovascular disease [58], which strongly influenced randomized trials [51, 54–57]. In infection, numerous studies (including by ourselves) have shown differential effects on the risk of infection with IL-6 inhibition [56]. MR studies show—in line with our understanding of biology and our clinical experience with IL-6 inhibition—an increased risk of the incidence of some infections (eg, skin and soft tissue infection, pneumonia) [56] but a reduced risk of severe infections (eg, intensive care admission with sepsis [57], critical COVID-19 [59]). This underscores that our outcome reflects not only acquisition of infection, but the progression of that disease. It is essentially certain that for many exposures causal effects may differ between various stages of infection and over the life course, and researchers should be careful in describing their results in this context.

Finally, one other example of a useful but difficult to interpret MR analysis in infection is performing MR of a causal risk factor for a disease where infection is known to be another risk factor: it is clear that Epstein-Barr virus (EBV) acquisition at a particular developmental age is the major cause of many cases of multiple sclerosis (MS) [60]. Vitamin D has also been long associated with MS but causality has now been shown convincingly in genetic studies [60]. However, randomized trials of vitamin D supplementation in those with MS have not yet been convincing [53, 61, 62]. What is vitamin D doing here? Is its effect on MS independent of EBV, or is it only relevant in patients who acquire EBV? Is it by modifying the host response at the time of infection or, possibly, priming early infection pathways and altering metabolic state but not being directly relevant at the time of infection? These are unanswered questions, but it is clear that in large cohorts where EBV status, immune response, and vitamin D levels are all measured at multiple time points, MR can unpick the confounding associated with observed vitamin D and answer causal questions.

## SUMMARY

MR can be a powerful technique helping identification of causal effects even in the presence of issues such as reverse causation and confounding that plague other observational studies. However, infection poses unique challenges for MR. Firstly, it requires the presence of a discrete exposure, which is often highly stratified by population, geography, and time. In the most extreme cases, this means MR can literally be impossible (eg, an MR of malaria exposure using outcomes in the United Kingdom). Secondly, defining infection clinically is in many cases challenging, and data on the causative pathogen is extremely rare in large datasets. In many cases, the diagnosis of infection is a combination of exposure and specific features (eg, severity) that lead to health care presentation. Proxies like antibody or cellular response are not generally easily interpretable exposure measures: a variant that increases cytomegalovirus antibody response would not tell you whether acquiring cytomegalovirus increases your risk of cardiovascular disease.A very careful understanding of what each exposure actually means, and whether the causal question is appropriate needs to be considered for all infection MR studies. Thirdly, many SNPs identified as strongly associated with infection are in the HLA region of the genome, which is challenging for MR (although methods are being developed [26]). Finally, MR of infection is also biased for any of the other reasons that MR can be biased, such as population stratification, pleiotropy, sample overlap, heritable confounding, etc. that are outside the scope of this article, but are likely present [7].

However, we should not be despondent. There are many applications of MR and similar approaches within infection. Firstly, with the increasing availability of genetic instruments for proteins and gene expression, drug target MR might identify potential causal targets, while other exposures with good genetic instruments (eg, iron status) are likely to answer questions of clinical importance [50, 53] . Secondly, the variability of pathogen presence and exposures could be used to help understand the causal effects of variants even when there is pleiotropy. For example, the difference in effects of the variant encoding sickle cell trait in populations with and without malaria [39] can inform on whether effects are likely secondary to malaria or the underlying genetic variation. That is, if the effects on say, anemia, are present in all populations, it is likely that the variant itself is causing anemia. If we only see an effect on anemia in populations where malaria is endemic, then we can be more confident that the effect of the variant is acting through reduction in malaria (ie, there is no violation in the third IV assumption) [39].

More complex designs that can consider time varying effects could be postulated: does the effect differ in populations that are exposed to a pathogen at a young age or later in life? If and when detailed clinical phenotyping becomes available, it may be possible to unpick any heterogenous effect of variants across the infection time course.

## CONCLUSION

MR can provide useful causal inference about the causes and consequences of infectious disease. However, in practice, many studies violate key assumptions of MR or downplay limitations of the technique. As large-scale human genetic data on infectious diseases becomes increasingly available, the use of MR is set to increase further, and researchers should be cognizant of its benefits and challenges.
